# Supplementary material for: Remote control of self-assembled microswimmers
Source: Sci Rep. 2015 Nov 5;5:16035. doi: 10.1038/srep16035 (PMC4633596; doi:10.1038/srep16035)
Supplement: Supplementary Information [file srep16035-s1.pdf]

April 29, 2015

Remote control of self-assembled microswimmers : Supplementary materials

G.Grosjean[a], G.Lagubeau, A.Darras, M.Hubert, G.Lumay, and N.Vandewalle
GRASP, Institute of Physics B5a, University of Liège, B4000 Liège, Belgium.

Supplementary information

Three movies accompany the paper :

- Movie 1 - Illustration of the experimental setup as well as the magnetocapillary interaction between particles.
- Movie 2 - Motion of an efficient microswimmer for the following experimental conditions : forcing frequency $f = 0.5\,\mathrm{Hz}$, vertical field $B_z = 30\,\mathrm{G}$, horizontal field amplitude $\beta_x = 10.4\,\mathrm{G}$, and offset $B_{x0} = 0.75\,\mathrm{G}$. The video is in real time.
- Movie 3 - An example of path control : the formation of the U-turn when the horizontal field is rotated step by step. Only one image per field oscillation is displayed. The video is accelerated 16 times.

[a] Correspondance : GRASP, Physics Department, University of Liège, B-4000 Liège, Belgium. http://www.grasp-lab.org
